# Alterations of Eye Movement Control in Neurodegenerative Movement Disorders

**DOI:** 10.1155/2014/658243

**Published:** 2014-05-18

**Authors:** Martin Gorges, Elmar H. Pinkhardt, Jan Kassubek

**Affiliations:** Department of Neurology, University of Ulm, Oberer Eselsberg 45, 89081 Ulm, Germany

## Abstract

The evolution of the fovea centralis, the most central part of the retina and the area of the highest visual accuracy, requires humans to shift their gaze rapidly (saccades) to bring some object of interest within the visual field onto the fovea. In addition, humans are equipped with the ability to rotate the eye ball continuously in a highly predicting manner (smooth pursuit) to hold a moving target steadily upon the retina. The functional deficits in neurodegenerative movement disorders (e.g., Parkinsonian syndromes) involve the basal ganglia that are critical in all aspects of movement control. Moreover, neocortical structures, the cerebellum, and the midbrain may become affected by the pathological process. A broad spectrum of eye movement alterations may result, comprising smooth pursuit disturbance (e.g., interrupting saccades), saccadic dysfunction (e.g., hypometric saccades), and abnormal attempted fixation (e.g., pathological nystagmus and square wave jerks). On clinical grounds, videooculography is a sensitive noninvasive *in vivo* technique to classify oculomotion function alterations. Eye movements are a valuable window into the integrity of central nervous system structures and their changes in defined neurodegenerative conditions, that is, the oculomotor nuclei in the brainstem together with their directly activating supranuclear centers and the basal ganglia as well as cortical areas of higher cognitive control of attention.

## 1. Introduction

Eye movement assessment potentially provides a valuable window into the human central nervous system function and may help to obtain insights into the structure of complex forms of human behavior including attentional control [1, 2]. Furthermore, the study of oculomotor control in pathological conditions offers insight into the underlying neural mechanisms. Neurodegenerative movement disorders are frequently accompanied by a broad spectrum of oculomotor abnormalities since large parts of the human central nervous system contribute to the function of “vision” comprising visual areas, oculomotor areas, and associated visual memory structures including eye movement control [3]. A large part of knowledge about these higher oculomotor functions results from electrophysiological investigations in the monkey brain and functional imaging in humans by use of advanced test paradigms which have shown eye movement-related activity in several cortical and subcortical areas [4–6]. In addition, computer-based neuroimaging such as fiber tracking by means of diffusion tensor imaging has depicted major pathways that are linked to oculomotor control and its changes in neurodegenerative movement disorders such as Huntington Disease (HD) [7, 8]. Oculomotor abnormalities in Parkinson's Disease (PD) have recently been reported to be associated with higher functional networks revealed by “task-free” intrinsic functional connectivity neuroimaging techniques [9]. The nature of the contribution of intrinsically interacting large-scale cortical functional networks to eye movements and their key to pathological dysfunction is currently being addressed in neuroimaging research.

Evidence for the need of intrinsically organized brain activity associated with visual input depends on the almost infinite visual information received by the human eye from the external environment [10]. Before guiding the eyes adequately in the orbit, a target of special interest in the visual scene needs to be determined, followed by target selection and by “programming” the oculomotor system in a coordinated manner to rotate the eye ball until the object is foveated. The entropy, a measure of information, of the visual stream arriving at the human eye is about 10^10^ bits/s whereas only approximately 3,000,000 bits/s are leaving the retina due to the limited number of available axons of the optic nerve and finally less than 10,000 bits/s are believed to be under attentive scrutiny [11]. This complex system covers most of the human brain specific characteristics. Thus, the investigation of eye movements has been applied as an experimental tool over the past four decades and provides a unique opportunity to understand the functional integrity of brain structures both in the healthy brain and in pathological state. The latter include the broad variety of Parkinsonian Syndromes [5] and other neurodegenerative conditions such as amyotrophic lateral sclerosis [12], fronto-temporal lobar degeneration [13], and Alzheimer's disease [14]. Pathological conditions are of special interest to ophthalmologists, neurologists, and scientists in order to get insights into potential alterations of the complex oculomotor networks, including fundamental issues of human behavior comprising conflict resolution and free will [2].

Retinal structure is divided into the fovea centralis with ultimate vision in its center and the larger periphery (with markedly less visual acuity) so that humans have developed the ability to foveate or refoveate an object of interest in the visual field. Rotating the eye ball offers a somewhat more economical strategy to shift or to maintain an object of interest on the fovea than turning the whole head [15]. In general, eye movements are required to compensate small head movements to sustain stability of gaze, to accurately track moving objects in the visual surrounding smoothly [16], or to rapidly redirect the eye onto a new target [17] rather than scanning the environment [15]. Eye movements can be subdivided into two main classes: one class of movements comprises vestibuloocular reflexes, optokinetic nystagmus, fixation, and smooth pursuit eye movements (SPEM) [3]. As the second kind of eye movements, humans use saccades (old French “saquer,” meaning “jerk”) in order to perform conjugated, fast eye movements shifting the eye ball discontinuously in a stepwise manner onto a new target [17].

This review summarizes the fundamental mechanisms of eye movement control, considering healthy and pathological states of the brain. More specifically, we discuss the main features of the oculomotor phenotypes that are specific for different movement disorders and that can serve as model conditions to study how distinct brain areas contribute to eye movement control. In addition, we focus on fixational eye movements as presenting a continuous range from microsaccades to (pathological) square wave jerks (SWJ) [18]. Due to the particular role of eye movement alterations for the clinical and neuroscientific work-up of Parkinsonian syndromes, we will focus on movement disorders in this synopsis to emphasize the significance in assessing eye movement control to understand the respective pathophysiology. Together, our aim is to condense both the oculomotor dysfunctions in patients with neurodegenerative movement disorders and the underlying pathological mechanisms that result in the observed dysfunctional oculomotor behavior. Beyond the fact that oculomotor dysfunctions can be important for the purposes of clinical diagnosis, we discuss the potential functions and mechanisms of higher cortical contributions to eye movement control, in particular by reviewing broad pathological spectrum of cognitive control in functional system-related neurodegenerative conditions.

## 2. Eye Movements during Attempted Visual Fixation in Health and Diseased States

During the absence of any (e.g., vestibuloocular) stimulus, healthy subjects are expected to withhold any unwanted considerable gaze shift maintaining the eyeball in its primary position. However, by performing attempted visual fixation of an unmoving target, conjugated small (<1°), jerk-like, involuntary saccades (microsaccades) and slow conjugated drifts are near-instantly observed [19]. For clear vision in the sense of the highest spatial resolution, the perception of an object should be optimized by small saccadic eye movements rather than by holding the image completely steady upon the retina. Perfectly fixed images upon the retina cause sensor adaption (fading) due to the property of being designed for the highest motion responsiveness like other sensory systems [20]. Thus, in case of attempted fixation of stationary targets, microsaccades play an important role in counteracting visual fading by shifting the image on the retina back and forth in small portions of approximately 0.5 degrees. In addition, larger voluntary saccades or blinks are considered to also effectively overcome visual fading [21]. The generation of microsaccades depends on the target size whereas luminance appears to be of no effect in modulating microsaccades [20]. The dynamics vary during scanning the visual scene, and the frequency of microsaccade production tends to increase with more challenging visual tasks which require increased visual discrimination abilities [22]. The relationship between microsaccade amplitudes with respect to their peak velocities follows the main sequence in an approximately linear manner [23]. Microsaccades can be suppressed by precision demanding tasks (e.g., threading a needle) but cannot be voluntarily evoked [24].

With respect to their amplitude, larger microsaccades are termed as SWJ and also become evident during fixation [18]. SWJ are thought to have the same neural substrate as microsaccades and present as conjugated, rapid eye movements (preferentially in horizontal direction) that intrude accurate fixation. More specifically, both microsaccades and SWJ continuously shift the eyes away from the target and back onto it in an involuntary manner [25]. In pathological conditions, they mostly present abnormally large and frequent. However, preschool children may present with larger SWJ (>1°) and less stable gaze holding [26] that rarely also occur in healthy adults but manifest more frequently in the elderly [19]. One diagnostic challenge rises from the large overlap of SWJ presentation between patients with movement disorders and healthy subjects [18].

Patients with neurodegenerative movement disorders frequently develop abnormal SWJ which frequently interrupt fixation, within the broad spectrum of oculomotor deficits [3, 27]. Since SWJ are believed to present as a continuous spectrum ranging from microsaccades to larger saccadic intrusions, the mechanisms generating SWJ appear to be similar to those of microsaccades and share a common oculomotor network with the saccadic system. In addition, it is relevant for the characteristics of SWJ in movement disorders that SWJ generation appears to be similar in the healthy brain and in pathological state. Larger SWJ probably reflect internal “neural noise” in the saccadic control loops and in the superior colliculus that forms a major component for the release of saccades by triggering the saccadic pulse generator in the midbrain [25]. The “neural noise” is hypothesized to initiate a saccade away from the target, resulting in a position error that is counteracted by shifting the gaze back onto the target. The higher this “neural noise” is (e.g., in movement disorders due to an impaired triggering of the superior colliculus from the basal ganglia), the more prominent SWJ seem to occur. The cerebellum may contribute to abnormal SWJ in addition [28] if it is involved in the pathological process in cerebellar diseases and neurodegenerative Parkinsonian syndromes other than PD.

During impaired stationary fixation such as sustained abnormal eye oscillations (e.g., large SWJ, pathological nystagmus), patients may report that vision is becoming subjectively blurred [21]. In general, two clinical approaches to abnormal fixation need to be distinguished, on the one hand, the examination of the patient's eye while the eye remains in primary position and on the other hand during the fixation in eccentric gaze holding [2]. In summary, abnormal oscillations of the eyes including pathological nystagmus and markedly large or frequent SWJ (beyond the aforementioned physiological fixation eye movements) account for a “red flag” symptom that should prompt further investigations with respect to differential diagnostic procedures [29]. The vast majority of these visual fixation signs are linked to dysfunctions of the central nervous system, in the absence of inaccurate vision, ophthalmologic diseases, or eye muscle affections.

## 3. Methods to Examine Eye Movements

Abnormalities during fixation can be investigated with Frenzel goggles or the ophthalmoscope by asking the patients either to hold their gaze steadily at the primary position or to shift their gaze towards eccentric positions. Frenzel goggles inspection of the patient's fixation ability accounts for a sensitive instrument to address fixational dysfunction or nystagmus. Unlike the ophthalmoscope, Frenzel goggles are equipped with small lights illuminating the patient's eyes and provide high-powered positive magnifying glasses (>+15 diopter) that disable the subject to adequately fixate any target in the visual field [30]. Hence, the examiner can detect abnormal SWJ and particularly pathological nystagmus since the absence of target fixation facilitates the manifestation of nystagmus forms that were attributed to peripheral vestibular impairment [31]. There are some drawbacks of these inspection methods. First, stimuli cannot be presented under specific conditions such as defined target positions. Second, the observations cannot be quantitatively characterized that is, metrics of saccadic accuracies, latencies with respect to stimulus onset, or determining peak eye velocities are not possible. The latter parameter is of special interest since peak eye velocity obtained during saccade performance characterizes the main sequence that provides robust metrics to assess pathology in peak eye velocity [17].

To overcome these limitations, computer-based recording techniques are applied to quantify subtle alterations in eye movement control, that is, electrooculography, scleral search coil systems, and videooculography (VOG). The past decades have emerged easy manageable computer-based eye trackers with integrated software environment for both stimulus design and automated data analysis for laboratory and portable usage. Scleral search coil systems and VOG emerge as the most widely used techniques to quantify eye movements, although electrooculography is yet the only device allowing recordings with closed eyes. The VOG measurement offers the best compromise between easily tolerable, noninvasive measurement and spatial and temporal resolution but requires advanced calibration techniques to be able to accurately quantify oculomotor performance. In contrast, scleral search coil systems provide optimum spatial and temporal resolution and warrant no calibration approach due to the absolute calibration by default [32] but are invasive since they are based on tightly fixated “contact lenses” carrying orthogonal coils. Another reason for VOG having become popular is the improved electronic hardware with additional software packages including both stimulus design and eye movement recording analysis features. Moreover, modern VOG systems provide easier usage and high portability with the possibility to assess human gaze behavior outside a dedicated room and even under dynamic conditions by utilizing an additional head-mounted camera (e.g., [33]). Video-based eye trackers comprising one or two head mounted and adjustable infrared miniature cameras allow online measurements so that the recorded data can be visually inspected in real-time. Commonly, the systems operate at about 250 Hz temporal sampling frequency which is constrained by spatial resolution of the field of view.

**Figure 1:**
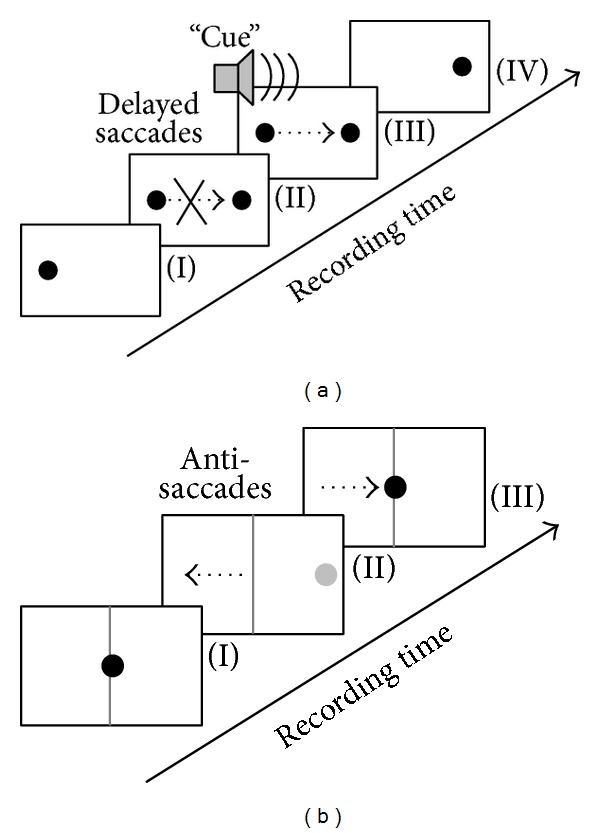
Illustration of cognitive demanding oculomotor tasks in order to assess attentional control. (a) In delayed saccades, subjects are asked to fixate the spot (I) and to withhold their gaze shift towards a new randomly appearing target (II) until an acoustic “cue” is sounded (III). The “run” is finished when the old target varnishes (IV). (b) The antisaccade task requires the subject to focus on the center position (I) until a new target (grey) presents randomly at either the right or the left eccentric position (II). The subject is immediately asked to shift the gaze to the contralateral half-plane (away from the target). The “run” is completed by shifting the gaze back onto the central position (III). Black dotted arrows illustrate the required gaze shift.

Basal oculomotor network function (at brainstem/basal ganglia/cerebellar level) is usually tested by visually guided reactive saccades [17, 34, 35]. In this paradigm, subjects are asked to track a randomly “jumping” target as quickly and as accurately as possible. Smooth pursuit eye movements are elicited by requiring the subjects either to track a continuously moving target [36] or to track a sinusoidally oscillating target [37]. In order to assess attentional eye movement control as a correlate of the cognitive (cortical) top-down oculomotor pathway, delayed saccades and antisaccades are executed. Both conditions aim at investigating the subject's ability to suppress the reflexive urge to shift their gaze towards a new suddenly upcoming target in the visual scene [38].  Figure 1 schematically illustrates two paradigms as an example for a cognitively demanding test.

## 4. Alterations of Eye Movement Control in Parkinsonian Syndromes

### 4.1. Parkinson's Disease (PD)

Parkinson's Disease (PD) is the second most common neurodegenerative disorder with cardinal motor symptoms comprising hypokinesia, tremor, rigidity, and postural impairment, while a multitude of nonmotor conditions including cognitive decline as part of the disease process has become evident [39, 40]. Autopsy-controlled studies by Braak and coworkers [41–44] indicated that the pathological process of PD can be characterized as a six-stage ascending spreading scheme beginning in the lower brainstem (stages 1-2) towards mesencephalic structures including the basal ganglia (stages 3-4) and finally reaching the cortex (stages 5-6). These findings suggest that PD has a preclinical stage and a symptomatic stage as soon as patients display the aforementioned cardinal motor symptoms defining clinical onset not before stage 3. Only a few studies have systematically investigated oculomotor dysfunctions in asymptomatic subjects with gene mutations. Whereas saccadic hypometria and the problems in withholding unwanted gaze shifts (hyperreflexivity) are a hallmark of both early PD and symptomatic PARKIN mutation carriers, presymptomatic PARKIN mutation carriers revealed undistinguishable oculomotor performance compared to age-matched healthy controls [45]. PD patients presented with a lack of attentional control resulting in the disability to withhold unwanted gaze shifts which appears to manifest even in nondemented PD patients [9]. This can be tested by utilizing tasks such as delayed saccades [9, 37, 46] or antisaccades [47].

PD onset typically incorporates motor symptoms caused by dopaminergic nigrostriatal cell degradation in the basal ganglia which are critical in locomotion including eye movements. The substantia nigra pars reticulata tonically inhibits the superior colliculus (SC) via GABA-ergic projections, whereas pausing the inhibitory SC input provides a prerequisite for saccadic release [48]. The SC is an important visuomotor structure and plays a major role in triggering both voluntary and reflexive saccades [49]. Moreover, the SC projects to the cerebellum via the nucleus reticularis tegmenti pontis. The cerebellum contributes to saccadic control in optimizing saccade trajectory by increasing eye acceleration during saccade onset and controls the movement procedure in order to keep the eye on track [50]. Cerebellar pathology in oculomotor function, however, typically cannot be observed in PD with very few exceptions (see below).

Unlike the basal ganglia, the SC remains intact until later stages in the pathological process [4], and both the SC and the striatum receive cortical input from the frontal eye fields (FEF), the supplementary eye fields, and the parietal eye fields [51]. Areas in the parietal cortex associated with oculomotor control beyond the parietal eye fields encompass superior and inferior parietal lobe and are a critical interface for attention and multiple sensory integration from visual and somatosensory modalities [52]. The supplementary eye fields contribute to target selection and visual search [53], and the FEF are critical in target selection of competing stimuli mediating their information to the SC and directly to the saccadic generator in the brainstem [54]. The striatum serving as the main input gate of the basal ganglia evaluates incoming and competing information for appropriate execution; however, with the putamen being the most affected area in PD, it remains to be discovered to what extent the PD pathology targets this mechanism [55]. The striatum gains also incoming streams from the dorsolateral prefrontal cortex which contributes to voluntary eye movements in the sense of inhibition control to prevent unwanted reflexive saccades [35]. As a part of the limbic system, the so-called cingulate eye field, located in the anterior cingulate cortex (which is involved in motivation, behavior, and executive control), contributes to saccade generation [56]. Guiding voluntary saccades requires several neural mechanisms within the framework of preemptive perception that manifests in activation in the cingulate eye field prior to the release of a saccade [55].

**Figure 2:**
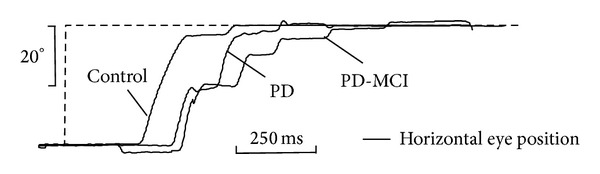
Visually guided horizontal reflexive saccade in a Parkinson's disease patient with mild cognitive impairment (PD-MCI) and a Parkinson's disease patient without cognitive impairment (PD), compared with an age-matched healthy control subject. Both PD patients presented with a considerable multistep sequence (saccadic hypometria) to shift their gaze onto the target (dashed-line). The videooculographically recorded data display the orthogonalized position for the cyclopean eye shown for the horizontal component (black lines). *x*-axis: acquisition time in seconds; *y*-axis: horizontal eye position in degrees.

Deterioration of dopaminergically mediated pathways in the basal ganglia in PD leads to overactive SC inhibition preventing the SC to trigger the brainstem saccadic generator. This may contribute to saccadic hypometria, as depicted in Figure 2, and slowed initiation of voluntary saccades [57] such as reduced number of rapid alternating self-paced saccades where subjects are asked to shift their gaze as fast and as accurately as possible between to stationary targets [45]. In PD, deep brain stimulation of the subthalamic nucleus has undoubtedly positive effects in treatment of motor symptoms [58], but the improvement of oculomotor performance is still debated. Compensatory effects on the functional level of the SC mediated by the substantia nigra pars reticulata have been reported [59] yielding improved saccade initiation and inhibitory control but did not significantly prevent prosaccades during antisaccade condition [60]. In contrast, Pinkhardt et al. [35] did not observe enhanced reflexive saccade performance. One possible explanation of these discrepancies could result from the included patients' degree of motor impairment. Since motor performance worsens in the course of PD, it could be hypothesized that those patients investigated by Yugeta et al. [60] presenting with a unified Parkinson's disease rating scale III score [61] in the ranges of 6–44 (stimulation OFF state) and 1–24 (stimulation ON state) were less severely affected than those reported by Pinkhardt et al. [35] with an UPDRS III score in the ranges of 16–64 (OFF) and 5–62 (ON). This may lead to the assumption that the less advanced patient group exhibited more benefits from deep brain stimulation on saccadic performance than those presenting with higher UPDRS III scores which is probably associated with cortical involvement. This hypothesis is in line with the findings of MacAskill and coworkers [4] who attributed oculomotor dysfunctions in early and noncognitively impaired PD to “pure” basal ganglia disorder whereas the more widespread cortical involvement later in the course of PD [44] may result in malfunctioning cortical areas involved in saccadic control.

Terao et al. [6] proposed a possible task-related modulation within the basal ganglia resulting in oscillatory spike activity that may contribute to both “hyperreflexivity” and slowed initiation. Moreover, functional connectivity neuroimaging revealed that connectivity loss in the putamen versus the caudate nucleus follows the same gradient as dopamine depletion, indicating a decoupling of the putamen prior to the caudate nucleus [62]. Other imaging studies on functional integration in PD patients [63, 64] indicated widespread functional remapping that likely alters connectivity associated with oculomotor function. The nature of the cortical contribution of large-scale higher function networks to oculomotor control remains a promising issue in future studies.

**Figure 3:**
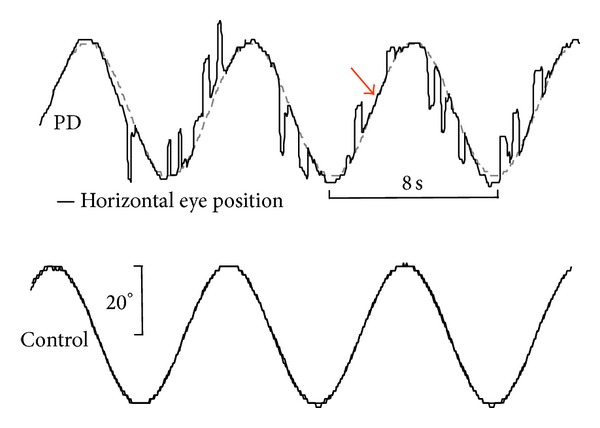
Horizontal smooth pursuit eye movements (SPEM) elicited by a sinusoidal oscillating spot (*f* = 0.125 Hz) and exemplified for a Parkinson's disease patient (PD, upper panel) and a representative age-matched healthy control (lower panel). The PD patient presents with severely affected SPEM, frequently interrupted by anticipatory saccades. Although SPEM is heavily impaired in PD, patients retained the ability to perform episodes of genuine smooth pursuit (arrow). For recording details, see Figure 2.

SPEM provide an optimum strategy of maintained movement adaptation in a highly predictive manner and involve large parts of the brain cortex, comprising primary visual areas like the striate and extrastriate cortex as well as the FEF and supplementary eye fields [16]. Likewise, the cerebellum is involved in performing pursuit and functions as a major hub after receiving the cortical efferents that are to be integrated in innervating the relays of the ocular motor neurons through the medial vestibular nucleus [50]. The easiest way to elicit SPEM is to ask a subject to track some continuously moving object in front of one's eyes. In the VOG, a sinusoidally oscillating target or an object moving with constant velocity can be presented. A quantitative measure of SPEM performance is the gain value describing the ratio of eye to target velocity. In patients with PD, SPEM are frequently interrupted or nearly abolished by anticipatory saccades resulting in a reduced pursuit gain [65], as depicted in Figure 3. This deficit already manifests early in PD and worsens with disease progression. Notably, even in advanced cases, the patients are fairly able to track the target smoothly whereas the episodes of performing SPEM exclusively shorten with more frequent saccadic intrusions [37]. Thus, the genuine SPEM system appears to be intact which raises the question of whether an executive dysfunction contributes to the characteristic anticipatory saccades during pursuit. For this fundamental issue, Pinkhardt et al. [35] suggested that accompanying extradopaminergic processes might cause SPEM impairment. Thus, a lack of inhibitory control which is closely linked to the higher functions located in the dorsolateral prefrontal cortex as well as the striatal projections [5] might explain these observations of dysfunctional SPEM. However, it remains an open issue to prove this hypothesis.

The mechanism of SWJ generation appears to be similar in PD patients and healthy controls, as indicated by Otero-Millan and coworkers [25]. Moreover, it was proposed that the characteristics of SWJ (such as frequency and amplitude) are linked to the internal neuronal noise level within the SC and the brainstem saccade generator. In pathological states such as PD, the saccade generator and the SC can be seen as a “neuronal noise amplifier” resulting in abnormal SWJ. In line with these findings, the SC might be triggered by an increase in FEF activity that compensates pathological increased inhibition of the SC from the substantia nigra pars reticulata [66].

In summary, PD patients present with a broad spectrum of disturbed oculomotor function comprising saccadic intrusions during SPEM, impaired inhibition control, and hypometric saccadic gains, while eye velocities used to be normal. Notably, these deficits manifest early in the disease course; however, they can be observed in the symptomatic rather than in presymptomatic stages of familial PD cases. In general, as the disease progresses, the oculomotor disturbances develop their full spectrum. The combination of both, the observed oculomotor phenotype and autopsy-controlled findings in PD, may increase our understanding of eye movement control as (i) the oculomotor nuclei in the brainstem appear to be spared by the PD-associated pathological process and (ii) the oculomotor deficits may primarily reflect a lack of attentional control. In the clinical context, the quantitative objective measure of eye movements by means of VOG has the potential as a possible technical surrogate marker in PD [6, 67].

### 4.2. Multiple System Atrophy (MSA)

MSA is a neurodegenerative disease characterized by autonomic and pyramidal dysfunction in addition to a broad spectrum of Parkinsonism presentations and cerebellar ataxia [68]. On neuropathological grounds, deterioration of nigrostriatal as well as olivopontocerebellar pathways contributes to the clinical phenotype of MSA [69] with the predominant Parkinsonian symptoms (MSA-P subtype) on the one hand and the predominant cerebellar dysfunction (MSA-C subtype) on the other hand. Nota bene, altered eye movements in MSA underlie both pathomechanisms [5]. In MSA-C, patients frequently present with typical cerebellar-type oculomotor signs comprising disturbed SPEM as well as downbeat, rebound, and gaze-evoked nystagmus [70]. Pathological nystagmus in the presence of Parkinsonism characterizes a unique identity for differentiating MSA from other Parkinsonian syndromes. In contrast, it is more difficult to distinguish MSA-P from PD because possible cerebellar symptoms mostly remain subtle; however, if present, they provide a “red flag” for MSA since cerebellar signs have not been reported in PD. Saccadic hypometria in MSA can be observed, with mildly or moderately inaccurate saccade amplitudes. MSA patients are principally able to generate normal saccade amplitudes, and peak eye velocities are unaffected in MSA compared to controls [37]. Reduced vertical eye velocities suggest in almost all cases a diagnosis different than MSA or PD. Most MSA patients present with abnormally large SWJ. Disruptions of SPEM as consecutive, fine-stepped catch-up saccades emerge predominantly in MSA-C, while patients with MSA-P can present with a mixed picture of both catch-up saccades and anticipatory saccades. The latter type cannot be distinguished from those observed in PD patients [37]. MSA pathology involves the brainstem nuclei associated with smooth pursuit eye movements. In addition, MSA patients present with the disability to withhold unwanted gaze shifts suggesting an impaired executive control [37] although MSA patients show cognitive deficits only in the late stages of the disease [5]. This aspect is worth mentioning since MSA patients may manifest, like PD patients, an attention deficit that can be discovered by cognitively demanding tasks in VOG (e.g., antisaccades, see Figure 1). These observations call for further investigations in order to study higher function networks that may contribute to the pathological process in MSA resulting in disturbed eye movement control.

### 4.3. Progressive Supranuclear Palsy (PSP)

Progressive supranuclear palsy (PSP) is characterized by Parkinsonism associated with signs like supranuclear gaze palsy, early falls, dysphagia, dysarthria, axially pronounced rigidity, and behavioral/cognitive impairment [71]. PSP can be subdivided into different subtypes that are characterized by their clinical course, most probably related to different patterns of pathological tau distribution in the brain. Apart from the “classical” PSP (Richardson Syndrome, PSP-RS), recent classifications subdivide clinical phenotypes including PSP-Parkinsonism (PSP-P), pure akinesia, progressive nonfluent aphasia, and corticobasal syndrome (CBS) [72, 73]. The eponymous supranuclear gaze palsy is a central element of all subtypes but is not present in all stages of all subtypes. The subtypes PSP-RS and PSP-P with the oculomotor hallmark of abnormally reduced vertical peak eye velocity are subsequently discussed. Oculomotor features might be diagnostically important as an early feature for PSP since definitive biomarkers remain to be defined yet, and subtle early clinical states of PSP may be indistinguishable from PD [74]. Slowing of saccades, particularly vertically, is caused by midbrain atrophy targeting burst neurons in the rostral interstitial nucleus of the medial longitudinal fasciculus that drives the extraocular eye muscles for vertical saccade generation [28]. Moreover, the omnipause neurons are required to suppress their firing while the burst neurons innervate the extraocular eye muscles to drive the saccade. A second inhibitory pathomechanism of the omnipause neurons is suggested to contribute to reduced peak eye velocity due to its interference with the burst neurons [5]. The dopaminergic nigrostriatal pathways and the superior cerebellar peduncle are reported to be involved in the pathological process resulting in prolonged latencies and (mostly subtle) cerebellar oculomotor signs, respectively [75].

**Figure 4:**
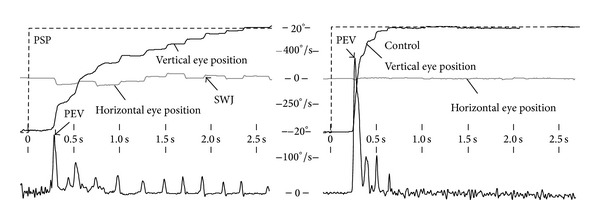
Videooculographic recordings depicting an upward visually guided reflexive saccade elicited in a sudden target jump ranging from −15 to +15 degrees in a patient with progressive supranuclear palsy (PSP, left panel) compared with an age-matched healthy control (right panel). The PSP patient reaches the target (dashed line) in a pathological multistep pattern, approximately 2.5 seconds (*x*-axis) after new stimulus appearance (black line, vertical gaze position, and *y*-axis) whereas the control's gaze shift is accomplished after about 600 milliseconds. Abnormal horizontal square wave jerks (SWJ) manifest in the orthogonalized horizontal eye position (gray line in the left panel), together with vertical saccades indicating a curved trajectory. In contrast, the horizontal eye position in the control subject (gray line in the right panel) exhibits no alteration. The lower row shows the corresponding vertical eye velocity (*y*-axis) computed by use of sample-by-sample differences of the vertical eye position signal. The PSP patient (left) fails in generating larger saccades resulting in a reduced peak eye velocity (PEV) compared with the control subject.

Study of oculomotor dysfunctions both in PSP-RS and in PSP-P revealed a similar presentation comprising slowed vertical saccades, saccadic hypometria, prolonged latencies, and impaired pursuit eye movement [65]. In advanced stages, PSP patients are fairly disabled to generate large saccade amplitudes, preferentially vertical, as exemplified in Figure 4. When vertical saccades become slowed, horizontal saccade velocity remains intact until the pathological process also involves horizontal burst neurons resulting in reduced horizontal peak eye velocities [3, 76]. PSP patients also present with disrupted visual fixation when they attempt to fix their eyes upon stationary targets. Additionally, they present with more frequent, larger SWJ (with amplitudes up to 5°), slower saccades, and more horizontal SWJ compared to controls [77]. The phenomenon of considerably higher prevalence of horizontal SWJ in combination with larger amplitudes may give a clue for PSP although microsaccades are observed preferentially in horizontal direction with increasing target size [24]. Two further explanations for the presence of abnormally large and frequent SWJ in PSP have been proposed: (i) since horizontal SWJ rate often increases during the release of vertical saccades, a SWJ coupling mechanism was suggested to enhance vertical saccade burst [13] and (ii) the decreased peak eye velocity and the resulting prolonged saccade duration may increase the probability that vision fades so that larger and more frequent SWJ could overcome visual fading in PSP [21, 28].

Remarkably, SPEM remain intact even in severely impaired PSP patients as long as a target is continuously moving in a predictive manner with low peak velocity and acceleration. This could be demonstrated when patients were asked to track a sinusoidal oscillating spot with low frequency [24]. With increasing stimulus frequency, the ability to perform SPEM considerably declines due to the disability to perform catch-up saccades to refoveate the target [37]. PSP is frequently accompanied by cognitive decline and frontal brain dysfunctions including executive deficits that can be demonstrated in cognitively demanding tasks such as the antisaccade condition in which the PSP patients often present with a limited ability to inhibit the visual “grasp” reflex in a sense of shifting the gaze towards the opposite target direction [28]. In addition, vergence eye movements are reported to be affected early in the PSP course and may also be associated with horizontal diplopia in some cases [78]. In summary, pathologically slowed vertical saccades' peak velocities are the eponymous hallmark of PSP. The PSP-associated damage involves midbrain structures including the saccadic burst generator in the brainstem that is responsible for the impaired (vertical) eye muscles innervation. Moreover, a hallmark of PSP is the early appearance of cognitive and behavioral deficits [79] that also manifest in oculomotor function by means of a considerable lack of inhibitory control of saccades (e.g., tested by antisaccades).

## 5. Alterations of Eye Movement Control in Huntington's Disease 

Autosomal dominant Huntington's disease (HD) is a progressive neurodegenerative disease, clinically presenting with a hyperkinetic movement disorder (chorea), cognitive decline, and behavioral symptoms [80]. The age of disease onset is predictable by the number of pathologically increased CAG repeats. Oculomotor deficits in patients with HD and presymptomatic gene carriers are reported to be one of the earliest signs [81]. They present as dysfunction of fixation ability [82], impaired initiation and inhibition of saccadic eye movements [83], impaired SPEM [2, 84], and decreased inhibition control in the sense of erroneously responding to novel stimuli [85–87]. Moreover, slowed saccades become prominent in both vertical and horizontal directions, latencies have been reported to be increased, and saccadic hypometria can be observed in HD like in other movement disorders [3]. Slowing of saccades is likely caused by midbrain atrophy, in particular in the pontine nuclei critical for the saccadic burst; however, the pathophysiology in oculomotor-related midbrain areas is ill-defined, so far [88].

Presymptomatic gene carriers show subtle cognitive and motor impairment due to striatal and cortical neuropathological changes that cause increased error rates during inhibition tests such as antisaccades [86] and likely reflect first clinical symptoms [7]. Reflexive saccades remain unaffected for a long time whereas both reflexive and voluntary-guided saccade performance decline with disease progression [89], since the structural connectivity between the frontal cortex and the caudate body seems to be particularly related to the control of voluntary-guided saccades [7, 86]. Difficulties in voluntarily initiating saccades in the presence of excessive saccadic intrusions during attempted fixation and a lack of inhibition control in the sense of withholding gaze shifts to new stimuli are apparently contradicting findings; a comprehensive explanation for this phenomenon in HD remains to be identified. HD-associated pathology appears to affect both the oculomotor nuclei “driving” the extraocular eye muscles and the attention system. The latter one is apparently involved already in presymptomatic HD.

## 6. Alterations of Eye Movement Control in Cerebellar Disorders 

Cerebellar signs manifest in many neurodegenerative movement disorders such as MSA and in the heterogeneous group of hereditary spinocerebellar ataxia. One prominent feature is cerebellar ataxia with impaired body posture; in addition, patients present with dysarthria, dysmetria, and dysdiadochokinesia [90]. Cerebellar dysfunctions in eye movement control frequently manifest in a variety of symptoms including the spectrum of pathological nystagmus, dysmetric saccades, abnormally large SWJ, postsaccadic drift as a consequence of pulse-step-mismatch, mildly slowed saccades, and a disturbed pursuit in the sense of corrective saccades interrupting SPEM [3, 16, 28, 37, 50, 88]. These deficits become pronounced in advanced cases, while many patients present with less dominant oculomotor abnormalities in early stages. In order to detect these symptoms, VOG is helpful beyond pure visual inspection. Oculomotor dysfunctions have been characterized by the genetically defined spinocerebellar ataxia subtypes [88]; for a comprehensive review, see [2]. Only a few studies investigated presymptomatic spinocerebellar ataxia gene carriers in contrast to HD. For spinocerebellar ataxia type 2 presymptomatic patients, a relation between CAG repeats, estimated time to disease onset, and decreased peak eye velocity has been reported [91]. Together, these VOG findings in cerebellar dysfunction mirror the cerebellar contribution to the oculomotor system, that is, refinement of saccade guidance, the adaptive strategy to perform perfect smooth pursuit, and the ability to hold the eye in a steady position. To our knowledge, the role of the cerebellum in attentional oculomotor control remains incompletely defined yet and might be a promising issue for future investigations.

## 7. Summary

In the absence of definitive biomarkers, VOG holds promise for a complementary noninvasive tool to characterize the oculomotor phenotype of distinct disease entities within the spectrum of neurodegenerative diseases. In the course of neurodegenerative disorders, disease-specific brain structures get systematically damaged. Hence, the resulting clinical condition might be considered as an investigational model for the contribution of functional components to eye movement control.* In vivo* examination of the oculomotor system offers a valuable window into altered brain function in the pathological state of movement disorders. Thus, we can learn about the contribution of different functional systems that may interfere with the way we direct our attention in the visual scene. In addition, oculomotor control covers large portions of the whole brain that appear to be decomposable into two major subdivisions: (i) the oculomotor nuclei responsible for the innervation of the six extraocular eye muscles and (ii) the much more complex network of higher cognitive control that is strongly associated with visual attention.

The investigation of eye movements may become important to clinicians in the context of differential diagnostics of movement disorders such as in distinguishing between Parkinsonian syndromes or to uncover a possible cerebellar contribution to pathological processes. VOG provides a sensitive noninvasive* in vivo* method to detect alterations in oculomotion function in patients with neurodegenerative movement disorders. Malfunctioning oculomotor control appears to have some characteristic feature that can give clues to be attributed uniquely to the subtype of the movement disorder. More specifically, other neurodegenerative types of Parkinsonian syndromes can be differentiated from PD early in the course. One should keep in mind that some of the Parkinsonian-associated hallmarks such as slowed eye velocities could also manifest in other neurodegenerative (nonmovement) disorders, resulting in the necessity for careful interpretation of VOG results in the light of the clinical presentation. Particular aspects such as SWJ or larger intruding eye movements may provide motivation for future investigations (possibly together with functional brain imaging studies [9, 92]) to increase our understanding of the functional pathoanatomy of these neurodegenerative conditions.

Notably, attentional dysfunction in oculomotor control mostly presents early in the course of neurodegenerative movement disorders even while no obvious cognitive deficits exist. This finding prompts the notion that even a subtle pathology of cortical networks may cause a broad variety of oculomotor alterations. To further investigate the complex nature of visual attention and the way we direct or withhold our gaze, it might be safe to assume that we can learn much from pathological conditions related to specific functional systems. This approach offers the possibility to refine our existing models of human oculomotor networks whose functional interaction may be considered an essential framework for higher functions such as visual attention.
